# Comparison of trabecular metal cups and titanium fiber-mesh cups in primary hip arthroplasty: A randomized RSA and bone mineral densitometry study of 50 hips

**DOI:** 10.3109/17453674.2011.572251

**Published:** 2011-04-05

**Authors:** Thomas Baad-Hansen, Søren Kold, Poul Torben Nielsen, Mogens Berg Laursen, Poul Hedevang Christensen, Kjeld Soballe

**Affiliations:** ^1^Department of Orthopaedic Surgery, Aarhus University Hospital; ^2^Northern Orthopaedic Division, Aalborg University Hospital, Denmark

## Abstract

**Background:**

Trabecular metal has shown promising results in experimental studies of bone ingrowth. Several clinical studies support these results. However, until now, no randomized clinical radiostereometric analysis (RSA) studies have been published. In this randomized RSA trial, we compared a new acetabular cup with a surface made of tantalum trabecular metal and a cup with a titanium fiber-mesh surface.

**Patients and methods:**

Between 2004 and 2006, we operated 60 patients with noninflammatory hip arthritis. The patients were randomized to receive either an uncemented cup with a titanium fiber-mesh surface (Trilogy cup) or a cup with a trabecular tantalum surface (Monoblock cup). After 2 years, 50 patients had completed the study. The primary endpoint was cup migration within the first 2 years after surgery; the secondary endpoints were change in bone mineral density and Harris hip score at 3 months.

**Results:**

Both cup types showed excellent fixation. RSA revealed minimal translation and rotation at 2 years. There was no statistically significant difference between the cup types with regard to translation. However, less rotation along the transverse axis was seen in the trabecular metal cups than in the fiber mesh cups: mean –0.01º (95% CI: –0.11 to 0.12) for trabecular metal cups and –0.60º (–0.72 to –0.48) for fiber-mesh cups (p = 0.04). The degree of periprosthetic bone loss was similar between the cup types in any of the regions of interest at 2 years of follow-up. 3 months postoperatively, we found a similar increase in Harris hip score in both groups: from around 50 to over 90.

**Interpretation:**

We found promising early results concerning fixation of trabecular metal components to the acetabular host bone. However, we recommend a longer observation period to evaluate the outcome of this new cup design.

Wear and implant loosening are the two most critical factors that influence the longevity of uncemented cups, and a prerequisite to achieving bone ingrowth is initial stability of the implant (Soballe et al. 1992). Clinical studies have shown good short-term and long-term results with porous coated cups in terms of wear (Gonzalez et al. 2004) and aseptic loosening (Clohisy and Harris 1999, Engh et al. 2004).

In contrast to femoral implants, only a few randomized clinical trials of uncemented cups using RSA have been reported (Karrholm and Snorrason 1992, Onsten et al. 1994, Thanner et al. 2000) because RSA of metal-backed cups is technically demanding (Baad-Hansen et al. 2007).

It has been hypothesized that the advantages of the Monoblock cup lie not only in the tantalum surface material but also in the cup design. In theory, the hemi-elliptic design of the Monoblock cup may increase the initial stability of the acetabular component, especially in the area of the rim (Sculco 2002). The metal-backed shell is made of trabecular metal, which consists of interconnecting pores, resulting in a structural biomaterial that is 75–80% porous. This allows a higher rate of bone ingrowth compared to conventional porous coatings, and increased interface shear strength (Bobyn et al. 1999a, b). In addition, due to a bone-matched elastic modulus of the trabecular metal, a reduction in stress shielding might be possible (Bobyn et al. 1999a) and a higher friction coefficient may improve primary implant fixation (Cohen 2002).

Our hypothesis was that a tantalum trabecular metal-surfaced acetabular cup would give a higher degree of fixation over a larger area, leading to better initial stability. We designed a randomized radiostereometric analysis (RSA) trial comparing the tantalum trabecular metal-surfaced acetabular cup with a titanium fiber-mesh surface cup. The primary endpoint was cup migration within the first 2 years after surgery as a predictor of early implant loosening (Kärrholm 1989). The secondary endpoints were change in bone mineral density and changes in Harris hip score.

## Patients and methods

The design and conduct of the clinical trail were approved by the local ethics committee (reg. no 20030159) before patients were included in the study. The procedures were in accordance with the ethics standards of the national ethics committee responsible and with the Helsinki Declaration (II). Additional permission was granted by the local ethics committee to perform 10 double RSA and dual-energy X-ray absorptiometry (dexa) examinations. The trial is registered at ClinicalTrials.gov (reg. no. NCT00116051).

Patients were included (1) if they had primary osteoarthritis, (2) if had sufficient bone density to allow uncemented implantation of an acetabular component, and (3) if they had no regular intake of non-steroid anti-inflammatory drugs (NSAIDs).

Suitable participants were recruited (60 patients, median age 62 (52–76) years, 34 men) and informed consent in writing was obtained from all patients at the outpatient clinic prior to surgery; detailed information was given by the operating surgeon and a project nurse.

At this time, the scheduled date of surgery was established. Follow-up at the intervals specified was performed by the operating surgeon and project nurses at outpatient examinations.

The participants were randomized to either a cementless implant with a titanium fiber-mesh surface (Trilogy cup; Zimmer, Warsaw, IN) or an implant with a trabecular tantalum surface (Monoblock cup; Zimmer). A computerized random number generator randomized the choice of acetabular component. No stratification with regard to sex, age, or disease stage was used. 60 sealed opaque envelopes containing the code were prepared. After commencing surgery, an envelope was drawn by a scrub nurse to decide which of the two cups should be used. On the femoral side, an uncemented Versys stem (Zimmer) was used. All patients received a 28-mm cobalt-chromium alloy head.

A senior surgeon (PHC) at Farsø Hospital, Farsø, Denmark, performed all the operations. A posterolateral approach was used. Progressively larger reamers were used to prepare the acetabulum. Both cups were implanted at approximately 45 degrees of abduction and 15 degrees of anteversion. The Monoblock cup chosen for implantation had the same size as the last-used reamer due to cup design, whereas the Trilogy cup was inserted with a 1–2 mm under-reaming technique.

### Cup design

**Figure 1.**
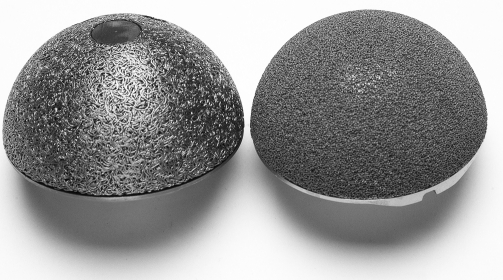
Trilogy cup (left) and Monoblock cup (right).

Both acetabular components evaluated were uncemented implants. The Trilogy cup is a modular hemispherical metal-backed cup consisting of a polyethylene liner and a metal shell (Figure 1).The liner used was a 10° liner of crosslinked polyethylene made of GUR 1050 resin, and sterilized by gamma irradiation in a nitrogen environment. The metal shell was a non-holed shell made of titanium-aluminum-vanadium alloy core upon which a fiber metal porous surface of pure titanium was fastened.

The Monoblock cup is hemi-elliptical (Figure 1). In the periphery, it has a built-in extension of 2 mm. The metal-backed shell, deposited upon a titanium alloy ring, is made of trabecular metal consisting of interconnecting pores. The cup is a non-modular system where the 10° liner is compression molded directly into the metal shell. Similarly to the Trilogy cup, the liner material is of crosslinked polyethylene consisting of GUR 1050 resin and is sterilized by means of gamma irradiation together with rest of the metal shell.

### Radiostereometric analysis (primary endpoint)

Intraoperatively, six to eight 1.0-mm tantalum markers were inserted in the periacetabular bone to form a reference rigid body. Radiostereometric examinations were done within the first week after surgery and at 3, 12, and 24 months after surgery. There was a calibration cage (RSA Biomedical, Umeå, Sweden) beneath the patient, holding 2 phosphor plates according to the uniplanar technique (Selvik 1990). 2 stationary radiographic tubes were placed in such a position that the X-ray beams would cross at an angle of approximately 40º. The exposure was set to 150 kV and 3 mAs.

All radiographs were digitized at 150 dpi using a Cobrascan CX 312T scanner, saved in a bitmap file format, and uploaded to a workstation. Model-based RSA (MbRSA) version 3.2 (Medis Specials, Leiden, the Netherlands) (Kaptein et al. 2003, 2004, 2005) was used to calculate the migration of the implant.

10 patients underwent double examinations to assess the precision of the RSA system. Between the 2 investigations, the equipment was removed from the location and repositioned before the second session within 5–10 min.

### Dual-energy X-ray absorptiometry (DEXA) (secondary endpoint)

Measurement of bone mineral density (BMD) was performed by DEXA with a Norland XR 36 Scanner (Norland Corporation, Fort Atkinson, WI). The patients were scanned shortly after the operation, and 1 and 2 years postoperatively. The scanner was calibrated on a daily basis according to the manufacturer's guidelines. Illuminatus DXA version 4.2 software (Norland Corporation) was used to define the regions of interest (ROIs). On each scan, the BMD was calculated for 4 ROIs according to Wilkinson et al. (2001).

### Statistics

We performed normal quantile plots to test data for normality. Repeated measurements analysis of variance (ANOVA) test was used for the statistical analysis. p-values of < 0.05 were considered to be statistically significant. To reduce the risk of type-I error (multiple comparison), a Tukey's HSD (honestly significant difference) test was applied in cases of statistically significant values.

Correlations between parameters were tested using Spearman's Rank Correlation.

The coefficient of repeatability (CR) was calculated as 1.96 times the standard deviation of the differences (d) between the 2 measurements, as a measurement of the precision of the system.

The power calculation for the primary outcome (RSA) was based on an estimated clinically significant difference of 0.6 mm in migration (Δ), and a standard deviation (SD) of 0.7 mm (Ryd. 1992) between groups. The prestudy sample size calculation required 22 patients (N) in each group to achieve 80% power (Cβ) at the 5% significance level (C2α): N = (C2α + Cβ)2 × SD2/Δ2. Due to the risk of patient dropout, 30 patients were included in each group. Statistical analysis was performed with the STATA 8 software package.

## Results

### Participant flow

**Figure 2.**
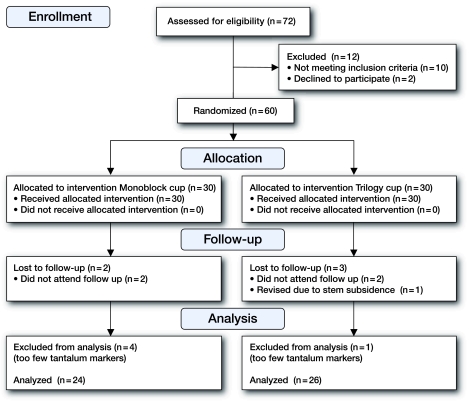
Flow chart of patient recruitment and randomisation strategy.

All operations were one-sided. 2 patients (2 hips) were excluded from the RSA because of an insufficient numbers of tantalum markers. 3 patients were excluded because of over-projection of acetabular bone markers. 4 patients did not attend the radiographic follow-up. 1 patient, randomized to the Trilogy cup group, was re-operated during the observation period because of a too small inserted implant leading to stem subsidence of the femoral component and was excluded even though the acetabular implant was left unchanged. In total, 10 patients were excluded; therefore, 50 patients were followed for 2 years (Figure 2).

### Radiostereometry (primary endpoint)

**Figure 3.**
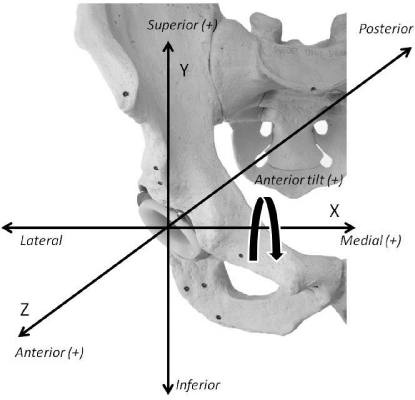
Cup rotation.

**Figure 4.**
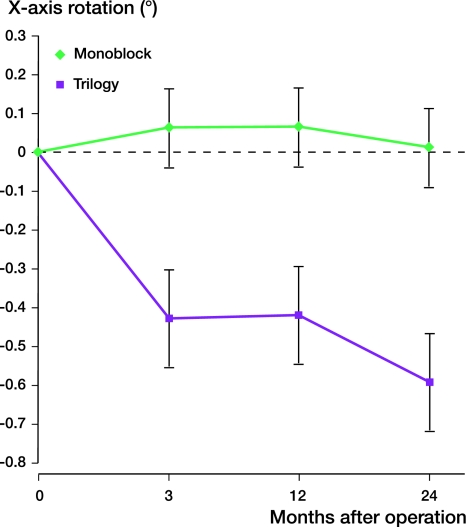
Rotation along the x-axis of both cup types over time. Values are mean and SD.

The precision was calculated from double examinations as described by Valstar et al. (2005) and expressed as 99% CI (Table 1). In both groups, the magnitude of translation was small in all 3 directions at 2 years, without any statistically significant difference between groups (Table 2). In terms of rotation, there was a statistically significant difference between groups along the transverse axis (Figures 3 and 4 and Table 2), mean –0.01 (95% CI: –0.11 to 0.12) for Monoblock and –0.60 (–0.72 to –0.48) for Trilogy (p = 0.04).

**Table 1.** Double examination of 10 patients. The precision is presented as mean + 2.7 SD of the error from the double examinations (99% confidence limits for statistically significant migration/rotation)

	Cup migration
Migration	
Medial-lateral (X)	0.11 mm
Proximal-distal (Y)	0.19 mm
Anterior-posterior (Z)	0.15 mm
Rotation	
Transverse axis (X)	0.33°
Longitudinal axis (Y)	0.35°
Sagittal axis (Z)	0.45°

**Table 2.** Migration 2 years postoperatively. Values are mean with 95% confidence intervals

	Monoblock (n = 24)	Trilogy (n = 26)	p-value **^a^**
Translation, mm			
Medial-lateral (x)	0.08 (−0.47 to 0.63)	0.13 (-0.24 to 0.51)	0.4
Proximal-distal (y)	0.08 (−0.22 to 0.38)	0.18 (-0.27 to 0.63)	0.2
Anterior-posterior (z)	0.06 (−0.20 to 0.32)	0.13 (-0.23 to 0.49)	0.3
Rotation, degrees			
Transverse axis (x)	−0.01 (−0.11 to 0.12)	−0.60 (−0.72 to −0.48)	0.04
Longitudinal axis (y)	0.12 (−0.44 to 0.68)	0.44 (−0.92 to 1.8)	0.5
Sagittal axis (z)	−0.30 (−1.86 to 1.3)	−0.11 (−0.86 to 1.6)	0.4

**^a^**ANOVA.

### Bone mineral density (secondary endpoint)

Reproducibility of BMD measurements is expressed as the coefficient of variation value, as previously described. The values of least statistically significant change for the specific ROIs were: 0.11 g/cm^2^ for ROI 1; 0.13 g/cm^2^ for ROI 2; 0.1 g/cm^2^ for ROI 3; and 0.11 g/cm^2^ for ROI 4.

A reduction in BMD in all 4 ROIs was seen for both implant types (Table 3) The highest rate of periprosthetic bone loss was observed during the first year. The BMD loss continued but diminished thereafter. At the 2-year follow-up, the acetabular bone—regardless of cup type—had the highest BMD loss in ROI 1 and 2. The estimated group difference showed narrow confidence intervals (Table 3), supporting the conclusion that there was similar bone loss with both cup types. A statistically significant positive correlation was found between body mass index (BMI) and change in BMD loss in ROI 3 (r = 0.6, p = 0.03), suggesting that patients with low BMI have a tendency to lose more periprosthetic bone.

**Table 3.** Change in BMD. Absolute values in g/cm^2^ (95% confidence intervals)

	Region of interest (ROI)			
Period (months)	1	2	3	4
Monoblock				
0–12	–0.16 (–0.22 to –0.10)	–0.16 (–0.24 to –0.08)	–0.09 (–0.15 to –0.03)	–0.07 (–0.11 to –0.04)
12–24	–0.04 (–0.11 to 0.07)	–0.10 (–0.21 to 0.02)	0.00(–0.05 to 0.06)	0.00 (–0.03 to 0.03)
Total loss at 24 months	–0.19 (–0.23 to 0.04)	–0.26 (–0.37 to –0.15)	–0.09 (–0.13 to 0.05)	–0.08 (–0.12 to 0.04
Trilogy				
0–12	–0.16 (–0.32 to 0.01)	–0.25 (–0.44 to –0.06)	–0.10 (–0.28 to 0.07)	–0.10 (–0.24 to 0.04)
12–24	–0.05 (–0.18 to 0.07)	–0.04 (–0.14 to 0.06)	–0.01 (–0.10 to 0.08)	–0.01 (–0.08 to 0.07)
Total loss at 24 months	–0.29 (–0.52 to –0.06)	–0.33 (–0.55 to –0.12)	–0.21 (–0.42 to 0.01)	–0.13 (–0.31 to 0.04)

### Harris hip score

Harris hip score increased similarly in both groups at the 3-month follow-up, from 50 (28–70) to 92 (76–100) in the Monoblock group and from 48 (34–64) to 95 (77–100) in the Trilogy group.

## Discussion

We found excellent fixation of both cup types at 2 years of follow-up. Migration analysis in earlier studies of the Monoblock cup assessed by the Ein-Bild-Röntgen-Analyse (EBRA) method revealed a mean total implant migration of 0.67 mm at 2 years and high initial stability (Kostakos et al. 2008). Our study confirms these findings with regard to migration, even though a direct comparison is not possible.

3 earlier publications on RSA studies have described the migration pattern of the Trilogy cup and a cup with a similar geometry and surface material, the Harris-Galante cup. One randomized study compared a ceramic-coated Trilogy cup with and without screw fixation (Thanner et al. 2000). Another study compared the Harris-Galante cup with and without ceramic coating (Thanner et al. 1999). These 2 studies could not show any effect of the use of screws to enhance early fixation with regard to migration or rotation at 2 years of follow-up. Likewise, no difference in migration between the coated and uncoated Harris-Galante cup was found. However, a reduction in rotation along the x-axis of the coated cup was found at the 2-year follow-up. The third publication, involving a long-term study with 12 years of follow-up of uncoated Harris-Galante cups (type I and II), showed a minimum of translation and no increase in cup translation over time (Rohrl et al. 2006).

Our 2-year follow-up results are similar to the results from Thanner et al. (2000) for the Trilogy cup. A minimal migration along the x- and z-axis and a slightly greater degree of migration along the y-axis was seen. The friction coefficient of trabecular metal on bone is approximately twice that of other porous coated surface materials (Macheras et al. 2008). This may explain the reduced rotation of the Monoblock cup compared to the Trilogy cup in our study.

The 2 cup designs may lead to different gap patterns between the implant and host bone. With regard to the Monoblock cup, a recent study of 86 implanted cups revealed gaps postoperatively in one third of the cases, mainly between the polar area of the cup and acetabular host bone (Macheras et al. 2006). However, the authors found that gaps of > 5 mm were almost non-existent 6 months after the operation (Macheras et al. 2006). A radiographic evaluation of 414 Monoblock cups 2 years after primary surgery showed no evidence of lysis or need for implant revision, even though 19% of the cups had a clear polar gap postoperatively (Gruen et al. 2005). This may have been due to the hemispherical design optimizing initial rim fixation, and later on the strong osteoconductive effect of the Monoblock cup may have compensated for the lack of polar apposition.

The results of other clinical studies support these encouraging results, showing no radiographic signs of osteolysis in an observation period of 8 to 10 years (Xenakis et al. 2008).

In contrast, postoperative gaps have been found to be present in AP- and lateral radiographs in the dome area of the non-coated Harris Galante cup in approximately 13% and 8%, respectively (Thanner et al. 1999), which indicate that the hemispherical shape of the Trilogy cup has a slightly better apposition at the polar area of the host bone.

The Monoblock cup is a non-modular system, where the polyethylene liner is compression molded directly into the metal shell. The Monoblock design eliminates the risk of backside wear (articulation between liner and metal shell) and reduces the production of wear particles. However, the non-modular system excludes the possibility of revision of liners in cases of breakage, or even more importantly, in the case of recurrent dislocations. Furthermore, screw augmentation to enhance initial stability is not an option when using the Monoblock cup.

We found promising results with regard to fixation of trabecular metal components to the acetabular host bone, but our study is limited to a 2-year follow up. Longer observation periods are necessary to evaluate the long-term migration pattern and clinical outcome for this specific implant.
